# Non-Canonical EZH2 Transcriptionally Activates RelB in Triple Negative Breast Cancer

**DOI:** 10.1371/journal.pone.0165005

**Published:** 2016-10-20

**Authors:** Cortney L. Lawrence, Albert S. Baldwin

**Affiliations:** Lineberger Comprehensive Cancer Center, University of North Carolina School of Medicine, Chapel Hill, North Carolina, United States of America; University of South Alabama Mitchell Cancer Institute, UNITED STATES

## Abstract

Enhancer of zeste homology 2 (EZH2) is the methyltransferase component of the polycomb repressive complex (PRC2) which represses gene transcription via histone H3 trimethylation at lysine 23 (H3K27me3). EZH2 activity has been linked with oncogenesis where it is thought to block expression of certain tumor suppressors. Relative to a role in cancer, EZH2 functions to promote self-renewal and has been shown to be important for the tumor-initiating cell (TIC) phenotype in breast cancer. Recently a non-canonical role for EZH2 has been identified where it promotes transcriptional activation of certain genes. Here we show that EZH2, through a methyltransferase-independent mechanism, promotes the transcriptional activation of the non-canonical NF-κB subunit RelB to drive self-renewal and the TIC phenotype of triple-negative breast cancer cells.

## Introduction

Studies have shown that a subpopulation of cells within a tumor exhibit the ability to self-renew and to initiate tumor formation, while the majority of the cells within the tumor are unable to self-renew and are poorly tumorigenic. These cancer stem cells (CSCs) or tumor initiating cells (TICs) are long-lived and have been shown to be resistant to chemotherapy and to promote tumor reoccurrence and metastasis [1,2]. Importantly, studies have shown that TICs are enriched in high grade tumors and are a predictor of poor prognosis [3]. TICs are observed in multiple subtypes of breast cancer [4,5], including Her2+ cancers and triple negative breast cancer (TNBC), an aggressive subtype of breast cancer characterized by the loss of estrogen receptor (ER), progesterone receptor (PR) and HER-2/neu [6]. Determining signaling pathways critical to TICs offers the potential for new treatments of cancer. In this regard, activities of the NF-κB transcription factor family as well as activities of the histone methyltransferase EZH2 have been shown to be important in self-renewal of triple-negative breast cancer (TNBC).

The NF-κB family consists of 5 proteins, p65 (RelA), RelB, c-Rel, p50/p105 and p52/p100, all sharing a common Rel homology domain [7]. Upon stimulation of cells with cytokines or other ligands, NF-κB becomes activated by one of two pathways. The canonical pathway consists of the activation of the canonical IκB kinase (IKK) complex, causing IKKβ to phosphorylate IκBα resulting in its degradation and the subsequent release of p65-p50 dimers. In the non-canonical pathway activated IKKα phosphorylates p100 resulting in proteolytic processing to p52 and release of p52-RelB dimers [7]. In the nucleus, NF-κB dimers activate the transcription of genes involved in cell cycle regulation, inflammation and suppression of apoptosis. Constitutively activated NF-κB can lead to oncogenic transformation through inflammation, cell proliferation, invasion and inhibition of apoptosis [8–11]. Furthermore, NF-κB has been shown to be important for the maintenance of breast TICs isolated from different subtypes of breast cancer including HER2+ [12,13] and TNBC [14]. It was shown that both canonical and non-canonical NF-κB promoted the TIC phenotype in triple-negative breast cancer cells [14]. Consistent with this, the NF-κB family of transcription factors has been shown to be activated in HER2+ and TNBC cells [14,15].

EZH2 is the catalytic subunit of the polycomb repressive complex 2 (PRC2) which represses gene transcription through the trimethylation of lysine 27 on histone H3 (H3K27me3) [16]. Upregulation of EZH2 levels has been associated with several cancers [16,17] promoting tumorigenesis partly through the silencing of critical target genes [18]. Additionally, EZH2 has been identified as a transcriptional activator of several genes in various cancers, but the mechanisms are less well characterized as compared to the role of EZH2 within the PCR2 complex. EZH2 was found to activate Notch1 and RAF1-β-catenin signaling in breast cancer to promote self-renewal [19,20]. In prostate cancer cells, the histone methyltransferase (HMT) activity of EZH2 is required in order to interact with androgen receptor and activate transcription, yet this is polycomb independent [21]. EZH2 promotes transcriptional activation of c-myc and cyclinD1 in conjunction with estrogen and WNT signaling and does not require its HMT activity [22]. EZH2 was shown to be important in SWI/SNF mutant cancers requiring both catalytic and non-catalytic functions [23]. Furthermore, EZH2 was reported to interact with the RelA/p65 and RelB subunits of NF-κB in TNBC to activate the transcription of several cytokines, and this was found to be independent of HMT activity [24]. Phosphorylation of EZH2 by CDK2 was shown to promote the oncogenic phenotype in TNBC [25]. Interestingly, TNBC tumors were shown to have elevated levels of EZH2 but surprisingly low levels of H3K27me3 [26] suggesting an HMT-independent role of EZH2 in this tumor subtype. Similarly, in ER- breast cancer cells RelB has been shown to be constitutively active since ER is known to inhibit RelB expression [27,28]. Recently, both EZH2 and RelB have been shown to play a role in the self-renewal of breast TICs [14,19,20] however the interdependence of these two pathways in the maintenance of TNBC TICs has not been explored. Our data identifies an HMT-independent role of EZH2 as a transcriptional activator of RelB in TNBC contributing to the maintenance of triple-negative breast cancer TICs.

## Materials and Methods

### Cell Culture and Reagents

SUM-149 cells were cultured in HuMEC supplemented with 5% fetal bovine serum (FBS) (Sigma), HuMEC supplement and bovine pituitary extract (Gibco). MDA-MB-231, MCF7 and 293T cells were cultured in Dulbecco’s modified Eagles medium (DMEM) (Gibco) with 10% FBS. HCC-1428 and T47D cells were cultured in RPMI (Gibco) supplemented with 10% FBS. All cell lines were obtained from ATCC. 3-Deazaneplanocin A (DZNep) was obtained from Sigma Aldrich. UNC1999 was a generous gift from the Jian Jin lab.

### siRNA Transfection

Small interfering RNAs (siRNA; siGenome SMART pool) against p65 (M-003533-02-0005), RelB (M-004767-02-0005), EZH2 (M-004218-03-0005), SUZ12 (M-006957-00-0005) and Non-Targeting #3 (D-001210-03-20) were purchased from GE Dharmacon. For tumorsphere assays cells were transfected with 50 nM siRNA with Dharmafect 1 reagent according to the manufacturer’s instructions. For all other studies, 25 nM siRNA was used. Samples were collected 72 hours post transfection for analysis.

### Tumorsphere Formation Assay

24 hours post siRNA transfection cells were plated for tumorsphere assays. Cells treated with UNC1999 were plated for the tumorsphere assay and treated with UNC1999 or vehicle for the first 3 days in culture. Cells were cultured under previously described conditions [14] and visually counted after a total of 7 days in culture.

### Immunoblotting

Cells were lysed in buffer containing 62.5 mmol/L Tris (pH 6.5), 5% glycerol, 2% SDS, 5% 2-mercaptoethanol, 1× protease inhibitor complete mixture (Roche Applied Science) and 1x phosphatase inhibitor cocktail 3 (Sigma-Aldrich). Protein concentrations were determined using Bradford assay against a standard of bovine serum albumin. Lysate protein (25 μg) was resolved by electrophoresis on SDS-polyacrylamide gels (Mini-PROTEAN TGX Precast Gels, Bio-Rad) and transferred to Immobilon-P membranes (Millipore). Membranes were blocked in 5% milk. Primary antibodies were p65 (8242), EZH2 (5246), SUZ12 (3737) and Actin (3700) from Cell Signaling Technology, RelB (sc-226) from Santa Cruz Biotechnology, H3K27me3 (07–449) from Millipore. Membranes were incubated with secondary antibodies conjugated to horseradish peroxidase (Promega) and complexes were detected using the Supersignal West Pico Chemiluminescent Substrate Kit (Pierce).

### Quantitative RT-PCR

Total RNA was isolated using TRIzol (Life Technologies). cDNA was synthesized from 1 μg of RNA using the iScript cDNA Synthesis Kit according to manufacturer’s protocol (Bio-Rad). Quantitative PCR was performed with diluted cDNA using Taqman Gene Expression Assay primer-probe sets from Applied Biosystems. Relative expression was normalized to glucuronidase beta using the ΔΔC_t_ method.

### Dual-Luciferase Reporter Assay

The RelB promoter containing 898 base pairs of sequence directly upstream of the start site was cloned into the PGL3-Basic vector (Promega) using SacI and XhoI restriction enzymes (New England Biolabs). 293 cells were transfected with 500 ng of the RelB reporter construct, 50 ng pRL-TK Renilla luciferase construct (Promega) and 500 ng of either pFLAG-CMV-p65 or pCMVHA-hEZH2 (Addgene, #24230) using polyethylenimine linear (Sigma-Aldrich). The cells were harvested 24 hours later in passive lysis buffer and analyzed according to Dual-Luciferase Assay System protocol (Promega). Activity was measured using an Lmax Luminometer (Molecular Devices) and relative light units were normalized to pRL-TK Renilla luciferase light units.

### Chromatin Immunoprecipitation Assay

The ChIP assay was adapted from [29] 2x10^6^ cells per immunoprecipitation were crosslinked with formaldehyde and the reaction was quenched by the addition of glycine. Cells were washed as previously described, lysed first in 5 mM PIPES pH 8, 85 mM KCl, 0.5% NP40, nuclei were collected and lysed in 50mM Tris pH 8, 10 mM EDTA pH 8.0, 1% SDS, then sonicated to yield DNA fragments less than 1000 base pairs using a Bioruptor from Diagenode. Aliquots corresponding to 2x10^6^ cells were diluted one fold in 2% Triton X-100, 300 mM NaCl, 1% Sodium Deoxycholate and precleared with a slurry of protein G-magnetic beads (Cell Signaling Technology), then incubated with 3 μg p65 (sc-109), RelB (sc-226) from Santa Cruz Biotechnology, EZH2 (39875) from Active Motif, SUZ12 (3737) from Cell Signaling Technology, H3K27me3 (07–449) or IgG (12–371) from Millipore. All solutions contained 1× protease inhibitor complete mixture (Roche Applied Science) and 1x phosphatase inhibitor cocktail 3 (Sigma-Aldrich). Antibody-protein-DNA complexes were captured with a slurry of protein G-magnetic beads. Beads were pelleted, and the supernatant from the IgG immunoprecipitation was used as input DNA. Beads were washed as previously described [29], the protein-DNA complexes were eluted in 2% SDS, 10 mM DTT, 0.1 M NaHCO_3_ and the cross-links were reversed by the addition of 0.2 M NaCl and incubation at 65° overnight. DNA was RNase and Proteinase K treated, phenol-chloroform extracted, ethanol-precipitated, and dissolved in TE.

### Quantitative PCR

Quantitative PCR (QPCR) was performed with 1μL of input DNA or ChIP DNA per reaction with Maxima SYBR Green/Rox (Fermentas). Absolute quantities were calculated using standards of input DNA. Primer sequences are RelB -631 5’-GGGTTACAACAACGCACAA-3’, RelB-529 5’CCTCCAAGGTCTCGCTAC-3’, MYT 5’-GCTGTGGGGAAAGGTAAGTC-3’, MYT 5’-ATGTCTCCTCTGTCAGACGC-3’.

## Results

### RelB and EZH2 Are Required for the Maintenance of TNBC TICs

**Fig 1:**
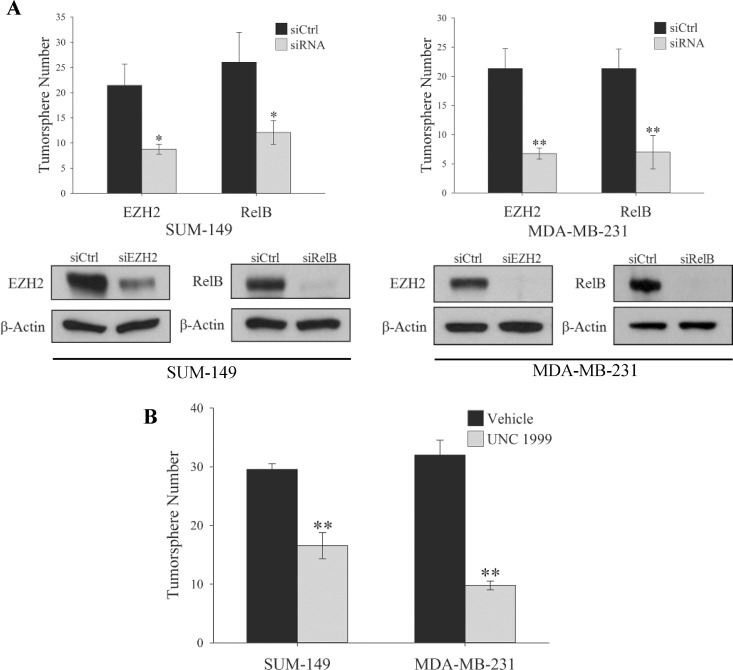
EZH2 and RelB are Required for the Maintenance of TNBC TIC. (A) Top panel, Quantification of tumorspheres formed by 100 SUM-149 or MDA-MB-231 cells expressing control siRNA or siRNA targeting EZH2 or RelB. Values given as means ± standard deviation from three biological repeats. *P<0.05, **P< 0.005 versus controls; two tailed unpaired t-test. Bottom panel, Immunoblots of indicated proteins in adherent SUM-149 or MDA-MB-231 cells expressing the indicated siRNA construct. β-actin serves as a loading control. (B) Quantification of tumorspheres formed by either 100 SUM-149 or MDA-MB-231 cells treated daily for 3 days with 1μM UNC1999. Values given as means ± standard deviation from three biological repeats. **P< 0.005 versus controls; two tailed unpaired t-test.

Previously our group showed that both canonical and non-canonical NF-κB signaling is required for the maintenance of TNBC TICs [14]. This data in combination with recent reports of EZH2 having a role in TNBC TIC maintenance [19,20] led us to examine the importance of both EZH2 and the non-canonical NF-κB subunit RelB in the self-renewal capacity of TNBC TICs. A hallmark of breast cancer TICs is the ability to form tumorspheres when plated at a low density in media containing no serum on low adhesion plates [30]. Knocking down RelB or EZH2 in both SUM-149 and MDA-MB-231 TNBC cell lines significantly reduced the number of tumorspheres formed (Fig 1A), as expected. Additionally, inhibiting the HMT activity of EZH2 with the small molecule, UNC1999 [31, 32], also reduced tumorsphere formation suggesting an HMT-dependent role of EZH2 in the maintenance of TNBC TICs (Fig 1B). Previous reports demonstrated that the HMT activity of EZH2 is not required in the expansion of stem cells in non-tumorigenic MCF10A cells [19] implicating two distinct roles of EZH2 in stem cell maintenance that potentially differs in tumorigenic versus non-tumorigenic cell lines.

**Fig 2:**
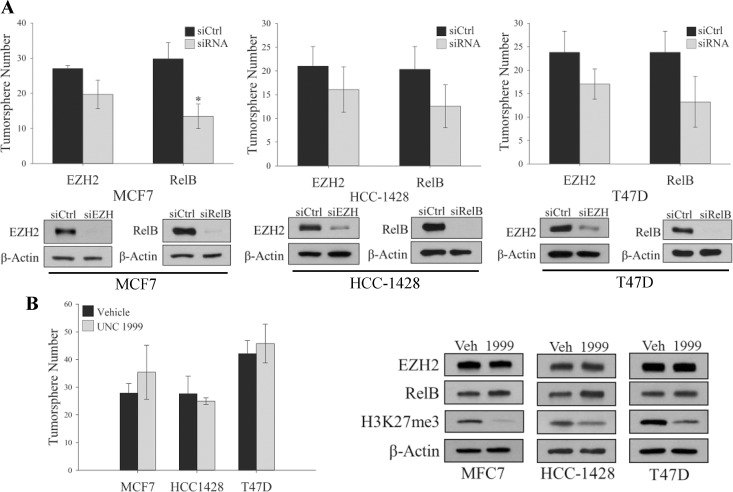
Self-renewal of TICs in ER+ Breast Cancer Cells is not Dependent on EZH2 and RelB. (A) Top Panel, Quantification of tumorspheres formed by 100 MCF7, HCC-1428 or T47D cells expressing control siRNA or siRNA targeting EZH2 or RelB. Values given as means ± standard deviation from three biological repeats. *P<0.05 versus controls; two tailed unpaired t-test. Bottom Panel, Immunoblots of indicated proteins in adherent MCF7, HCC-1428 or T47D cells expressing indicted siRNA construct. β-actin serves as a loading control. (B) Left panel, quantification of tumorpsheres formed by 100 MCF7, HCC-1428 or T47D cells treated daily for 3 days with 1μM UNC1999. Values given as means ± standard deviation from three biological repeats. Right panel, immunoblots of the indicated proteins in adherent MCF7, HCC-1428 or T47D cells treated daily for 3 days with 1μM UNC1999 to validate EZH2 inhibition. β-actin serves as a loading control.

In order to determine if these effects are unique to the triple negative subtype of breast cancer, the contribution of RelB and EZH2 to the self-renewal capacity of several ER+ cell lines was tested. Knockdown of EZH2 only weakly reduced self-renewal of these cells (Fig 2A), while RelB knockdown had a more pronounced response. Additionally, treatment of these ER+ cell lines with EZH2 inhibitor UNC1999 had no effect on tumorsphere formation (Fig 2B), thereby demonstrating that the HMT activity of EZH2 is not required for the maintenance of tumorspheres in some ER+ breast cancer cells.

### EZH2 Is a Transcriptional Activator of RelB

**Fig 3:**
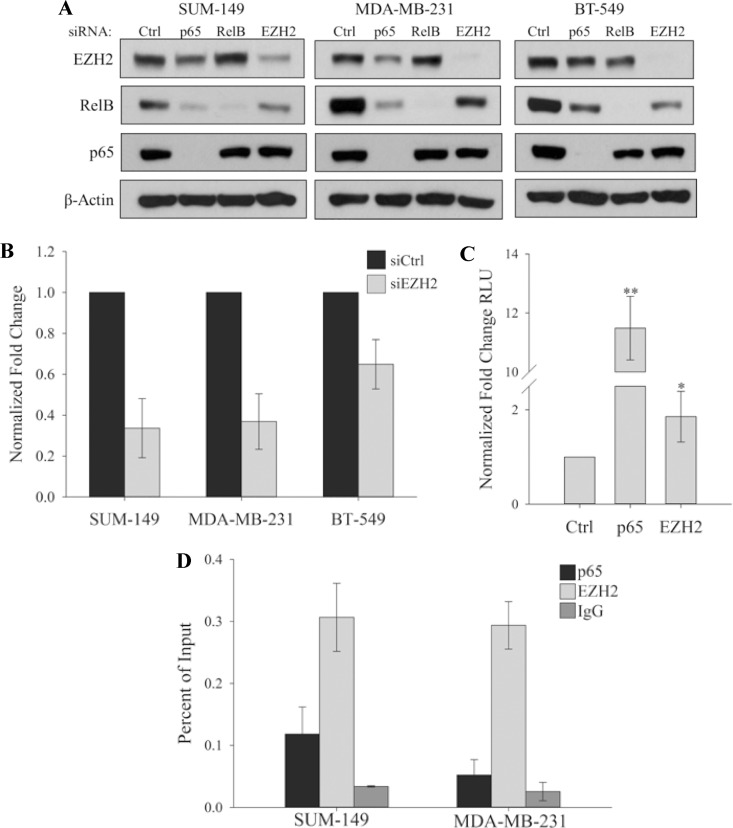
EZH2 is a transcriptional activator of RelB. (A) Immunoblots of indicated proteins in SUM-149, MDA-MB-231 or BT-549 cells expressing control siRNA or siRNA targeting p65, RelB or EZH2. β-actin serves as a loading control. (B) Real time PCR showing expression of RelB in SUM-149, MDA-MB-231 and BT-549 cells. Expression was normalized to glucuronidase beta. Values given as means ± standard deviation from three biological repeats. (C) Dual luciferase assay from HEK293 cells co-expressing the RelB Reporter with either Flag-p65 or HA-EZH2 and Renilla luciferase. Relative light units (RLU) was normalized to Renilla luciferase light units and expressed as fold over the RelB promoter alone. Values given as means ± standard deviation of four biological repeats. *P = 0.05, **P< 0.05 versus controls; two tailed unpaired t-test. (D) ChIP analysis of p65 and EZH2 binding at the RelB promoter in SUM-149 and MDA-MB-231 cells. Non-specific binding was estimated from IgG immunoprecipitates. Values given as means ± standard deviation from three technical repeats of one representative experiment from the three biological repeats.

The role of EZH2 as a transcriptional activator is less well characterized than its role as a transcriptional repressor in the PRC2 complex. It has been previously reported that EZH2 forms a complex with p65 and RelB in TNBC to regulate the transcription of a group of cytokines [3]. To potentially link EZH2 with RelB, we found that, in three different TNBC cell lines, the protein levels of RelB strongly decreases when EZH2 is depleted with either siRNA or shRNA (Fig 3A, and see S1 Fig). RelB levels also decrease after knockdown of p65, which is consistent with the ability of p65 to regulate RelB expression [33]. Importantly, RelB RNA levels decreased with EZH2 knockdown (Fig 3B) indicating transcriptional control of RelB by EZH2. To further address the potential for EZH2 to transcriptionally activate RelB, cells were transfected with p65 or EZH2 and with a reporter containing the RelB promoter. Luciferase activity increased with p65 or EZH2 to a statistically significant level (Fig 3C), suggesting that both p65 and EZH2 can activate the transcription of RelB. Consistent with this, ChIP analysis of the RelB promoter in TNBC cells demonstrated an enrichment of both EZH2 and p65 (Fig 3D) thereby providing further evidence that EZH2 is a transcriptional activator of RelB in TNBC.

### The Histone Methyltransferase Activity of EZH2 Is Dispensable for the Regulation of RelB Transcription

**Fig 4:**
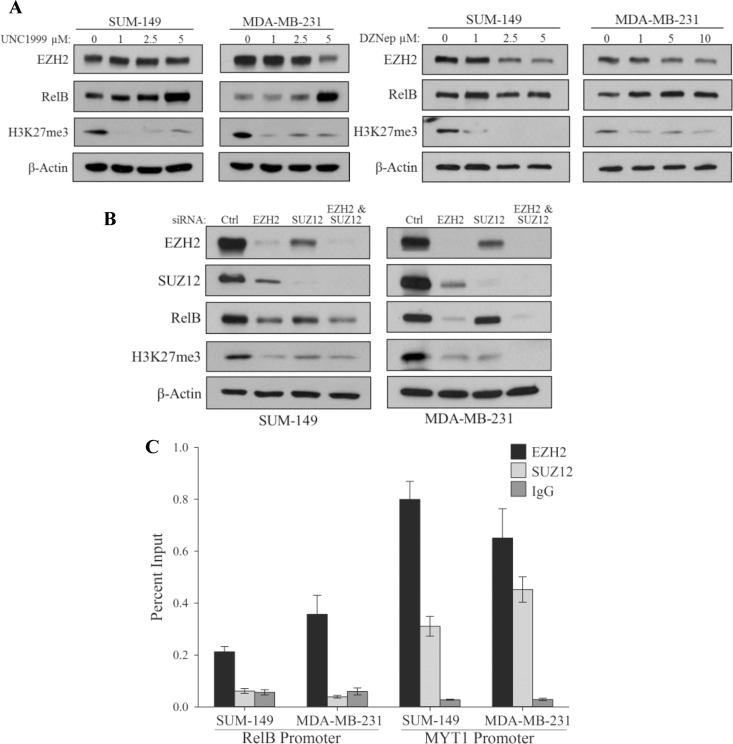
The HMT activity of EZH2 is not Required for RelB Transcription. (A) Immunoblots of indicated proteins in SUM-149 or MDA-MB-231 cells treated with the indicated concentrations of UNC1999 (left panel) or DZNep (right panel) for 72 hours. β-actin serves as a loading control. (B) Immunoblots of indicated proteins in SUM-149 or MDA-MB-231 cells expressing control siRNA or siRNA targeting EZH2, SUZ12 or both EZH2 and SUZ12. β-actin serves as a loading control. (C) ChIP analysis of EZH2 and SUZ12 binding at the RelB promoter or the MYT1 Promoter as a positive control in SUM-149 and MDA-MB-231 cells. Non-specific binding was estimated from IgG immunoprecipitates. Values given as means ± standard deviation from three technical repeats of one representative experiment from the three biological repeats.

It was important to determine if the methyltransferase activity of EZH2 is required for RelB transcription in TNBC. EZH2 was inhibited with small molecule inhibitors UNC1999 or DZNep, which blocked histone methyltransferase activity of EZH2 as detected by a decrease in H3K27me3 levels. Interestingly, this did not lead to a decrease in RelB levels (Fig 4A). To further validate that the regulation of RelB by EZH2 is PCR2-independent, we knocked down SUZ12, the subunit of the PCR2 complex which bridges the interaction of EZH2 to nucleosomes and is required for EZH2 catalytic activity [16]. As expected SUZ12 reduced EZH2 levels. Interestingly, RelB levels were elevated over the RelB levels observed upon knocking down EZH2 suggesting that the EZH2 pool remaining after knocking down SUZ12 is able to contribute to RelB transcription (Fig 4B). The histone methylation mark that EZH2 lays down, H3K27me3, was inhibited upon SUZ12 siRNA further indicating that the HMT activity of EZH2 is not required in regulation of RelB. To further validate that EZH2 is acting independent of the PRC2 complex, enrichment of SUZ12 at the RelB promoter was determined. Although EZH2 was again found to bind at the RelB promoter in TNBC cells, there was no enrichment of SUZ12 at this promoter (Fig 4C). The MYT1 promoter was shown to be regulated by the PRC2 complex [34] and was therefore used as a positive control. Indeed there was significant enrichment of EZH2 and SUZ12 in both TNBC cell lines (Fig 4C) further indicating that EZH2 is binding the RelB promoter and activating its transcription independent of the PRC2 complex.

## Discussion

Our studies demonstrate that the non-canonical NF-κB subunit RelB is transcriptionally regulated by EZH2 in TNBC partly contributing to the maintenance of CSCs. Previously it was shown that RelB [14] and EZH2 [19,20] are important for the self-renewal of breast cancer TICs. Here we demonstrate a mechanism linking these previous observations. EZH2 has been identified as a transcriptional activator in TNBC regulating the expression of Notch [19], as well as IL-6 and TNF [24]. In the latter study, regulation of IL6 and TNF was proposed to involve EZH2 in complex with RelB and p65. We showed that IL6 promotes the formation of TNBC TICs and that exogenous IL6 rescues tumorsphere formation when NF-κB is inhibited [14]. With our finding that EZH2 is a transcriptional activator of RelB it is likely that the upregulation of RelB by EZH2 feeds into the previously described pathway targeting IL-6 (and potentially other cytokines), to drive self-renewal. Since knockdown of p65 was shown to promote TNBC self-renewal and since p65 promotes RelB expression with EZH2, it is possible that a primary role for p65 in self-renewal is through the cooperation with EZH2, to promote RelB expression.

Our studies determined that the histone methyltransferase activity of EZH2 is not required for the regulation of RelB transcription, yet tumorsphere number decreases when treated with inhibitors of EZH2 catalytic activity suggesting a role for the histone methyltransferase activity of EZH2 in tumorsphere maintenance. We speculate that the HMT-dependent activity of EZH2 might involve STAT3 signaling. It has been shown that EZH2 methylates STAT3 in glioblastoma stem-like cells which is required for the activation of STAT3-dependent activation of stem cell-associated transcriptional factors and efficient tumorsphere formation [35]. Additionally, the JAK2/STAT3/IL6 pathway has been shown to be required for the growth of CD44-CD24+ breast cancer cells [36], cell surface markers believed to be associated with breast TICs [30]. This suggests a possible role for EZH2 in promoting breast cancer stem cells through the methylation and activation of STAT3. Interestingly, this pathway could be preferential for the triple negative subtype since UNC1999 treatment does not appear to affect tumorsphere formation in ER+ breast cancer cell lines possibly identifying a pathway specific to the aggressive TNBC subtype which could be therapeutically targeted through inhibition of EZH2 activity.

RelB was originally identified to function primarily in the regulation of adaptive immune response [37] and the involvement of RelB in tumorigenesis was less well characterized. RelB was shown to promote tumorigenicity of prostate cancer cells partly due to regulating IL8 levels [38]. Importantly, in prostate cancer tissue nuclear RelB was observed in more samples than p65 and there was a significant correlation between nuclear RelB and patient Gleason score [39]. Similarly, high nuclear levels of active RelB were found in carcinogen-induced murine mammary tumors [40] and nuclear RelB was found to be significantly elevated in nuclei from ER- inflammatory breast cancer tissue samples [41]. Additionally, inhibition of RelB with vitamin D3 sensitized breast cancers to ionizing radiation [42]. A signature gene set has been identified to be regulated by EZH2 and NF-κB specifically in TNBC [24]. Patients with high expression of this gene set were shown to have lower brain and lung metastasis-free survival emphasizing the importance of this signaling pathway in TNBC. The data reported here provide data linking the non-canonical functions of EZH2 with control of RelB, to drive self-renewal of TNBC cells. This mechanism may underlie key aspects of the oncogenic functions for EZH2 and RelB.

## Supporting Information

S1 Fig RelB Levels Decrease after Knocking Down EZH2 with shRNA. Immunoblots of the indicated proteins in SUM-149 and MDA-MB-231 cells after transduction with a lentivirus carrying pLKO.1-shScramble or pLKO.1-shEZH2 construct. β-actin serves as a loading control.
(TIF)
