# Supplementary figures and images for: Non-Canonical EZH2 Transcriptionally Activates RelB in Triple Negative Breast Cancer
Source: PLoS One. 2016 Oct 20;11(10):e0165005. doi: 10.1371/journal.pone.0165005 (PMC5072726; doi:10.1371/journal.pone.0165005)

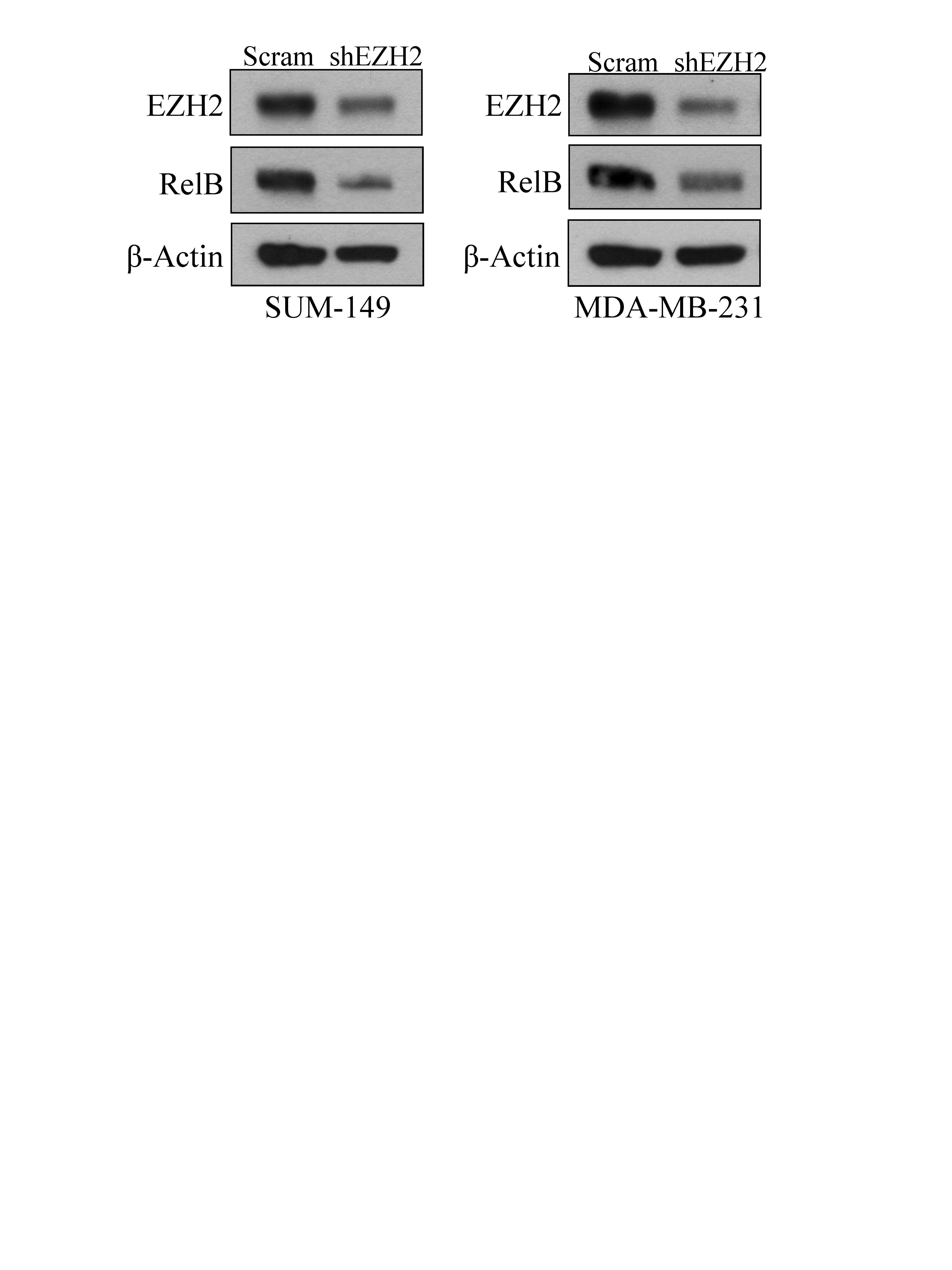

Supplement: S1 Fig — Immunoblots of the indicated proteins in SUM-149 and MDA-MB-231 cells after transduction with a lentivirus carrying pLKO.1-shScramble or pLKO.1-shEZH2 construct. β-actin serves as a loading control. (TIF) [file pone.0165005.s001.tif]
